# Activation of PPARγ regulates M1/M2 macrophage polarization and attenuates dextran sulfate sodium salt‐induced inflammatory bowel disease via the STAT‐1/STAT‐6 pathway

**DOI:** 10.1002/kjm2.12927

**Published:** 2024-12-30

**Authors:** Liang Xue, Yong‐You Wu

**Affiliations:** ^1^ Department of Gastrointestinal Surgery The Second Affiliated Hospital of Soochow University Suzhou China; ^2^ Department of General Surgery The First People's Hospital of Lianyungang Lianyungang China

**Keywords:** inflammatory bowel disease, macrophage polarization, PPARγ, STAT‐1/STAT‐6 pathway

## Abstract

This study aimed to investigate whether activation of PPARγ regulates M1/M2 macrophage polarization to attenuate dextran sulfate sodium salt (DSS)‐induced inflammatory bowel disease (IBD) via the STAT‐1/STAT‐6 pathway in vivo and in vitro. We first examined the effect of PPARγ on macrophage polarization in LPS/IFN‐γ‐treated M1 RAW264.7 cells and IL‐4/IL‐13‐treated M2 RAW264.7 cells. Then, 40 male C57BL/6 mice were randomly divided into five groups: the Sham, IBD, IBD + fludarabine (FLU), IBD + IL‐4, and IBD + pioglitazone (PI) groups. The mice received 2.5% DSS in their drinking water for 7 days and then received regular water for 2 days to establish the experimental IBD murine model. The mice in the IBD + FLU, IBD + IL‐4, and IBD + PI groups were intraperitoneally injected with FLU, IL‐4, and PI, respectively, for 9 days. Clinical symptoms, intestinal barrier function, macrophage polarization, PPARγ, and the STAT‐1/STAT‐6 pathway were analyzed. Activation of PPARγ decreased M1 polarization marker expression and STAT‐1 phosphorylation and increased M2 polarization marker expression and STAT‐6 phosphorylation in RAW264.7 cells. Activation of PPARγ attenuated disease symptoms, such as weight loss, diarrhea, and bloody stool. Histological analysis revealed that PI treatment reduced inflammatory cell infiltration, restored the mucosal architecture, and improved the expression of tight junction proteins. Moreover, the activation of PPARγ decreased the expression of iNOS and increased the expression of Arg‐1, Fizz 1, and Ym 1 by inhibiting STAT‐1 phosphorylation and promoting STAT‐6 phosphorylation in mice with DSS‐induced IBD. Activation of PPARγ regulates M1/M2 macrophage polarization to attenuate DSS‐induced IBD via the STAT‐1/STAT‐6 pathway in vivo and in vitro.

## 1 INTRODUCTION

Inflammatory bowel disease (IBD) is a complex group of multiple chronic, nonspecific intestinal inflammatory diseases that include Crohn's disease and ulcerative colitis. The intestinal inflammation that is caused by IBD can lead to digestive system symptoms, such as abdominal pain, diarrhea, and hematochezia. These symptoms often recur and seriously affect a patient's daily diet and nutrient absorption, which may lead to weight loss, anemia, and malnutrition.^1^ In addition, long‐term intestinal inflammation may increase the risk of acute abdomen, such as intestinal perforation and intestinal obstruction, which require emergency medical intervention.^2^ Currently, the cause of IBD is still not fully understood, but IBD is generally considered to be the result of the combined effects of genetic, environmental, infectious, and immune factors.^3^ These factors lead to abnormal activation of the intestinal immune system, which in turn triggers chronic inflammation of the intestinal mucosa.

As an important part of the immune system, macrophages play key roles in maintaining intestinal homeostasis and responding to foreign pathogen invasion.^4^ According to their functional status, macrophages can be divided into two main polarized types: classically activated (M1) and alternatively activated (M2) macrophages. M1 macrophages are involved in the proinflammatory response and release large amounts of inflammatory cytokines, such as TNF‐α, IL‐1β, and IL‐6, which lead to inflammation and injury of the intestinal mucosa.^5^ In contrast, M2 macrophages are involved in anti‐inflammatory and tissue repair processes. M2 macrophages release anti‐inflammatory cytokines such as IL‐10 and TGF‐β to inhibit excessive inflammation and promote the repair of the intestinal mucosa.^6^ Transcription factors play key roles in regulating macrophage polarization. The transcription factors associated with M1 macrophages include STAT‐1, NF‐κB, and IRF5.^7^, ^8^, ^9^ IFN‐γ and LPS can activate the STAT‐1 pathway and promote the M1 polarization of macrophages.^7^ In contrast, STAT‐6, C/EBPβ, and IRF4 are associated with M2 macrophage polarization, and IL‐4/IL‐13 can activate the STAT‐6 pathway and promote the M2 polarization of macrophages.^10^

During the pathogenesis of IBD, an imbalance in the polarization of macrophages is one of the important causes of disease progression as well as a reason that IBD is difficult to cure. When abnormal changes occur in the intestinal environment, such as an imbalance in the intestinal flora and damage to intestinal mucosal barrier function, macrophages may become excessively activated and polarized toward the M1 type, thereby releasing large amounts of inflammatory cytokines and exacerbating inflammation in the intestinal mucosa.^11^ This continuous inflammation not only leads to intestinal mucosal damage and dysfunction but also may further affect the balance of the intestinal microbiota, forming a vicious cycle.

As a nuclear receptor transcription factor, peroxisome proliferator‐activated receptor γ (PPARγ) not only plays important roles in adipocyte differentiation, lipid metabolism, and insulin resistance but also is extensively involved in the regulation of inflammation.^12^ PPARγ is an important target of anti‐inflammatory drugs, such as 5‐aminosalicylic acid and corticosteroids.^13^ The expression of PPARγ is reduced in the colonic epithelium of patients with ulcerative colitis, which may be an important factor leading to intestinal dysfunction and chronic inflammation.^14^ Similarly, animal experiments revealed that gut microbiota‐derived inosine in dietary barley leaf supplement attenuates colitis via activation of PPARγ.^15^ Activation of PPARγ by octanoic acid‐rich enteral nutrition attenuates sepsis‐induced ileum and colon injury via the STAT‐1 pathway.^12^

Therefore, we investigated whether activation of PPARγ regulates M1/M2 macrophage polarization to attenuate dextran sulfate sodium salt (DSS)‐induced IBD via the STAT‐1/STAT‐6 pathway in vivo and in vitro.

## 2 MATERIALS AND METHODS

### 2.1 Chemicals and reagents

DSS was obtained from MP Biomedicals (USA). Fludarabine (FLU), IL‐4, IL‐13, and IFNγ were obtained from MedChemExpress (Shanghai, China). Pioglitazone (PI) and SR202 were obtained from APExBIO (USA). Lipopolysaccharide (LPS) was obtained from Sigma‐Aldrich (USA). PPARγ and iNOS antibodies were obtained from Abcam (Cambridge, UK). Arg‐1, p‐STAT‐1, STAT‐1, p‐STAT‐6, STAT‐6, occludin, F4/80, and β‐Actin antibodies were obtained from Cell Signaling Technology (USA). The ZO‐1 antibody was obtained from Proteintech (USA).

### 2.2 Cell culture and treatment

RAW264.7 cells were purchased from Abbkine Scientific Co., Ltd. The cells were cultured in DMEM supplemented with 10% FBS and 1% penicillin at 37°C in 5% CO_2_. LPS (15 ng/mL) and IFN‐γ (50 ng/mL) were added to induce M1 macrophage polarization. IL‐4 (25 mg/mL) and IL‐13 (25 mg/mL) were added to induce M2 macrophage polarization. PI (10 μM) was added to increase the expression of PPARγ. SR202 (0.4 mM) was added to inhibit the expression of PPARγ. SR‐202 is a potent and specific PPARγ antagonist that can selectively inhibit thiazolidinedione‐induced PPARγ transcriptional activity and does not affect the basal or ligand‐stimulated transcriptional activity of PPARα or PPARβ.^16^ RAW264.7 cells were incubated with LPS/IFN‐γ, IL‐4/IL‐13, PI, or SR202 for 24 h and then collected to measure protein and mRNA expression.

### 2.3 Animal experiments

Six‐ to eight‐week‐old male C57BL/6 mice weighing 18.9 to 21.7 g were obtained from Soochow University. All the mice were housed in an SPF environment and were fed a standard diet with free access to drinking water. The procedure was performed with approval and following the guidelines of the Animal Experimentation Medical Ethics Committee at Soochow University.

The mice were randomly divided into five groups: the Sham, IBD, IBD + FLU, IBD + IL‐4, and IBD + PI groups (*n* = 8 per group). The mice received 2.5% DSS in their drinking water for 7 days and then received regular water for 2 days to establish the experimental IBD murine model.^17^ The mice in the IBD + FLU, IBD + IL‐4, and IBD + PI groups were intraperitoneally injected with FLU (20 mg/kg, every other day), IL‐4 (5 μg/kg/d), and PI (20 mg/kg/d), respectively, for 9 days. PI, FLU, and IL‐4 were dissolved in a vehicle consisting of 5% DMSO, 40% PEG300, 5% Tween 80, and 50% ddH_2_O. The dosages of PI, FLU, and IL‐4 that were used were based on previous studies.^12^, ^18^, ^19^, ^20^, ^21^ The mice in the Sham group were intraperitoneally injected with the same volume of vehicle solution and received drinking water and a standard diet for 9 days. Each mouse was kept in a separate cage, and changes in body weight, stool consistency, and hematochezia were observed daily. The disease activity index (DAI) was calculated on the basis of changes in body weight, stool consistency, and hematochezia from Days 0 to 9.^22^ The mice were anesthetized with pentobarbital sodium on Day 10. The entire colon was collected, and the length of the colon was measured.

### 2.4 Hematoxylin–eosin (H&E) staining

The colon was fixed in 10% buffered neutral formalin overnight and then embedded in paraffin. The colon was cut into slices (5 μm thick) and stained with hematoxylin and eosin. Changes in the mucosal architecture, including leukocyte infiltration, mucosal thickening, and epithelial cell erosion, were measured under a light microscope.

### 2.5 Periodic acid Schiff (PAS) staining

Colonic sections were treated with periodate solution (10 min) and stained with Schiff dye (15 min). The sections were subsequently washed three times with distilled water and stained with hematoxylin (3 min). Images of the sections were captured under an ordinary light microscope.

### 2.6 Immunofluorescence

Colonic sections were incubated with anti‐F4/80 (1:200), anti‐Arg‐1 (1:100), and anti‐iNOS (1:100) antibody solutions overnight. The sections were subsequently washed three times with PBS. The secondary antibodies were labeled with a fluorescent dye and used to enhance the fluorescence signal. Images of the sections were captured under a fluorescence microscope.

### 2.7 Immunohistochemical staining

The colonic sections were dewaxed, rehydrated, and subjected to antigen retrieval and then incubated with 3% hydrogen peroxide to block endogenous peroxidase activity. The sections were subsequently incubated at 4°C with anti‐ZO‐1 and anti‐occludin antibodies (1:100, overnight), followed by incubation with a biotinylated secondary antibody for 1 h at room temperature. Images of the sections were captured under an ordinary light microscope.

### 2.8 Real‐time polymerase chain reaction

Total RNA was extracted from the colons and RAW264.7 cells via TRIzol reagent. Upon separation, 1 μg of total RNA was reverse transcribed to complementary DNA. Real‐time PCR was used to measure complementary DNA expression with the following primers: Arg‐1: 5′‐CTCCAAGCCAAAGTCCTTAGAG‐3′ (forward) and 5′‐GGAGCTGTCATTAGGGACATCA‐3′ (reverse); Fizz 1: 5′‐CCAATCCAGCTAACTATCCCTCC‐3′ (forward) and 5′‐ACCCAGTAGCAGTCATCCCA‐3′ (reverse); Ym 1: 5′‐CAGGTCTGGCAATTCTTCTGAA‐3′ (forward) and 5′‐GTCTTGCTCATGTGTGTAAGTGA‐3′ (reverse); iNOS: 5′‐GGAGTGACGGCAAACATGACT‐3′ (forward) and 5′‐TCGATGCACAACTGGGTGAAC‐3′ (reverse); and β‐Actin: 5′‐ACAGAGCCTCGCCTTTGCCGAT‐3′ (forward) and 5′‐GACCCATGCCCACCATCACGC‐3′ (reverse). The expression of each mRNA was normalized to that of β‐Actin.

### 2.9 Western blotting analysis

Total protein was extracted from the colons and RAW264.7 cells via RIPA lysis buffer containing phosphatase inhibitors and protease inhibitors. Total protein was loaded onto 10% SDS–PAGE gels, separated (120 V for 90 min), and transferred to nitrocellulose membranes (300 mA for 90 min). The membranes were blocked with 5% nonfat dry milk and incubated overnight with primary antibodies against ZO‐1 (1:1000), occludin (1:1000), PPARγ (1:1000), p‐STAT‐1 (1:1000), STAT‐1 (1:1000), iNOS (1:1000), p‐STAT‐6 (1:1000), STAT‐6 (1:1000), Arg‐1 (1:1000), and β‐Actin (1:5000). The membranes were washed with TBST and then incubated for 1 h at room temperature with secondary antibodies (peroxidase‐conjugated goat anti‐rat IgG, 1:5000), and the optical density of the bands on their membranes was measured via a computer‐based imaging system.

### 2.10 Statistical analysis

GraphPad Prism 5 was used to analyze the data. The data are presented as the mean ± standard error of the mean. One‐way analysis of variance followed by Tukey's test was used for comparisons among multiple groups. A value of *p* < 0.05 was considered to indicate a statistically significant difference.

## 3 RESULTS

### 3.1 PPARγ regulated M1/M2 macrophage polarization via the STAT‐1/STAT‐6 pathway in vitro

**FIGURE 1:**
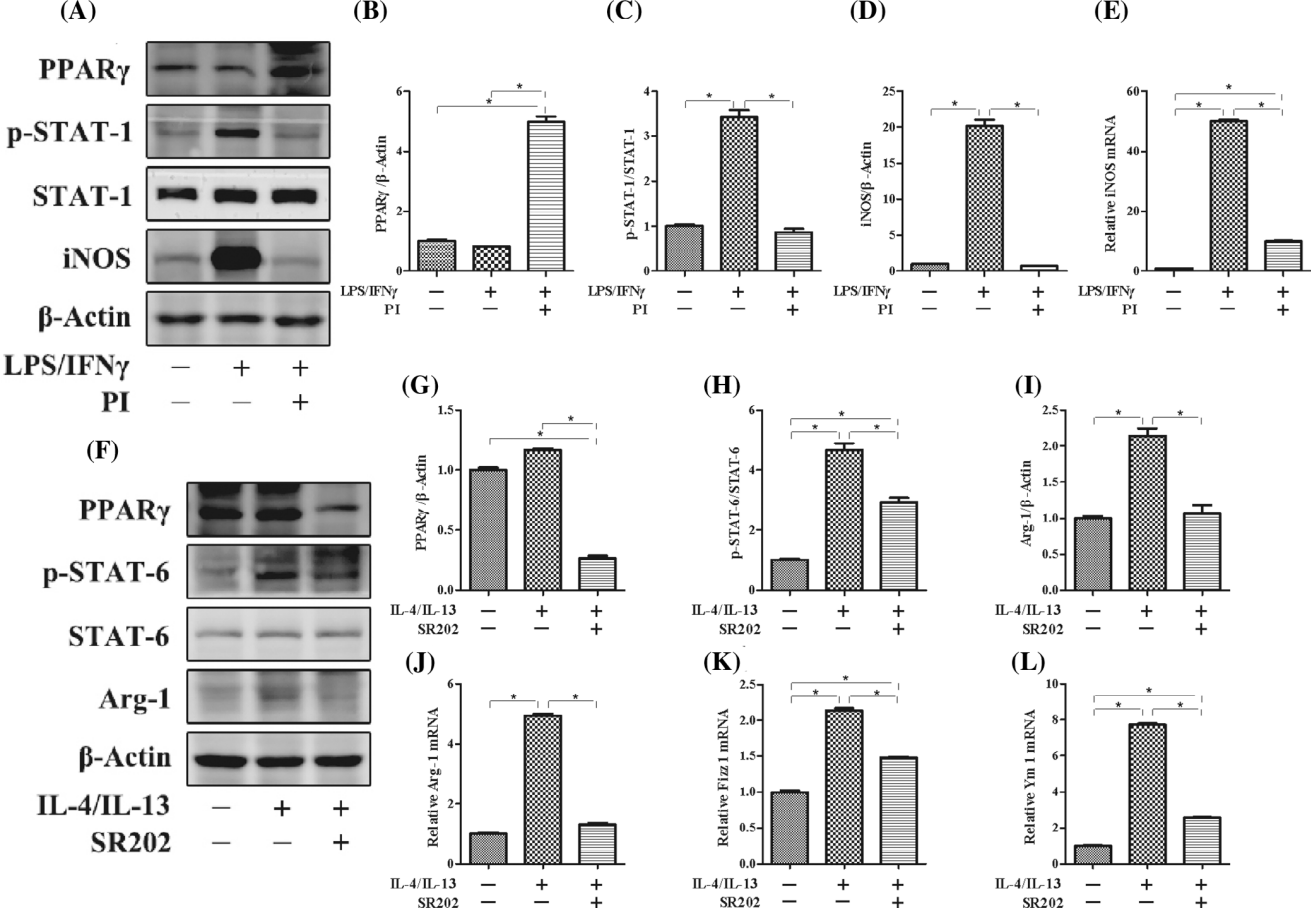
The influence of PPARγ on the polarization of macrophage in vitro. The protein expression of PPARγ, p‐STAT‐1, STAT‐1, iNOS (A), PPARγ, p‐STAT‐6, STAT‐6, and Arg‐1 (F) was measured by western blotting. The relative protein expression of PPARγ (B), iNOS (D), PPARγ (G), and Arg‐1 (I) was normalized to that of β‐Actin. The protein expression of p‐STAT‐1 (C) was normailzed to that of STAT‐1. The protein expression of p‐STAT‐6 (E) was normailzed to that of STAT‐6. The mRNA expression of iNOS (E), Arg‐1 (J), Fizz 1 (K), and Ym 1 (L) was measured by real‐time polymerase chain reaction. The expression of each mRNA was normalized to that of β‐Actin. The data are presented as the mean ± standard error of the mean. Significant differences are indicated as *p < 0.05.

**FIGURE 2:**
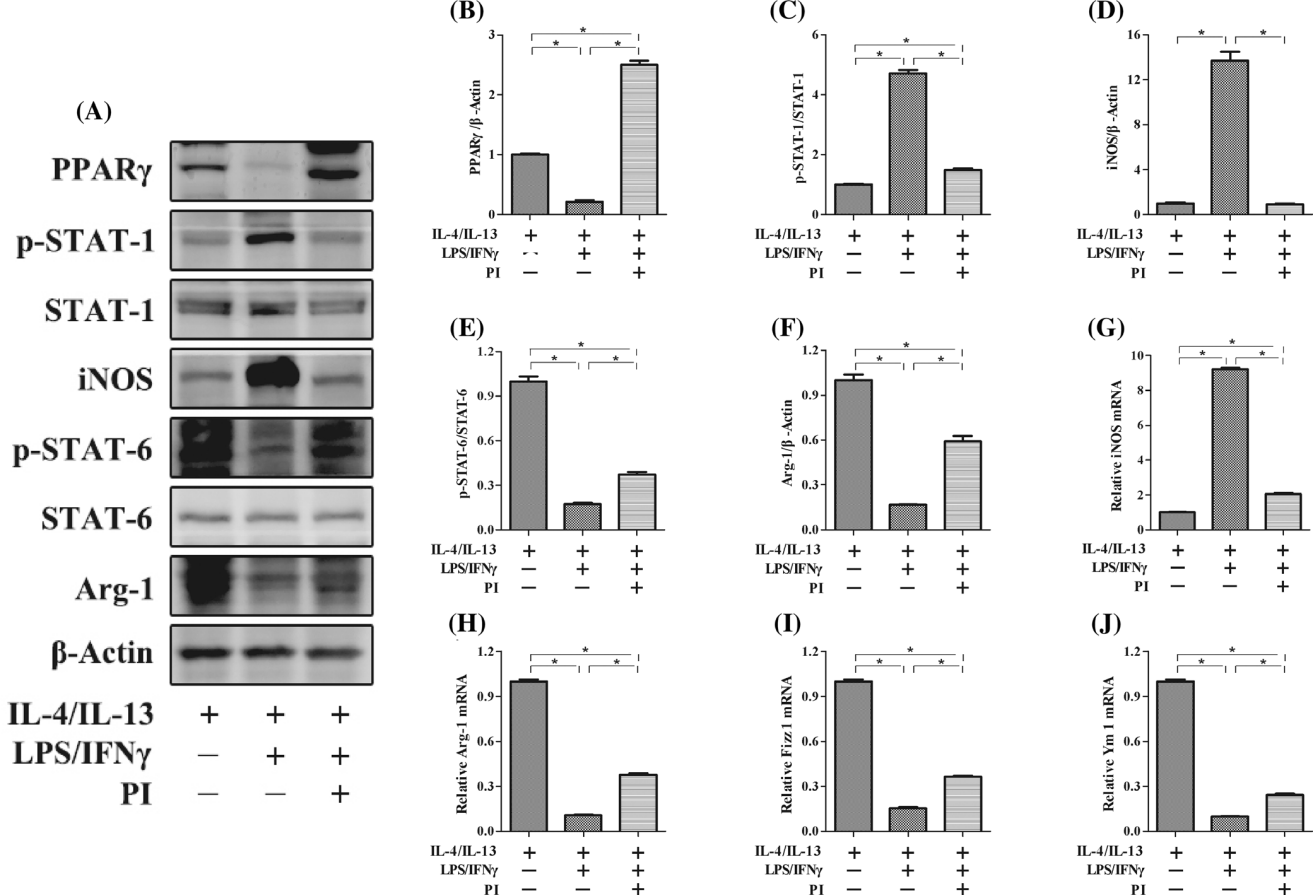
PPARγ regulates M1/M2 macrophage polarization through the STAT‐1/STAT‐6 pathway in vitro. The protein expression of PPARγ, p‐STAT‐1, STAT‐1, iNOS, PPARγ, p‐STAT‐6, STAT‐6, and Arg‐1 (A) was measured by western blotting. The relative protein expression of PPARγ (B), iNOS (D), and Arg‐1 (F) was normalized to that of β‐Actin. The protein expression of p‐STAT‐1 (C) was normailzed to that of STAT‐1. The protein expression of p‐STAT‐6 (E) was normailzed to that of STAT‐6. The mRNA expression of iNOS (G), Arg‐1 (H), Fizz 1 (I), and Ym 1 (J) was measured by real‐time polymerase chain reaction. The expression of each mRNA was normalized to that of β‐Actin. The data are presented as the mean ± standard error of the mean. Significant differences are indicated as *p < 0.05.

To investigate the potential mechanism by which PPARγ regulates macrophage polarization, we performed an experiment using the RAW264.7 macrophage line in vitro. First, LPS/IFN‐γ was added to induce M1 macrophage polarization. Stimulation with LPS/IFN‐γ significantly increased the expression of iNOS and STAT‐1 phosphorylation. However, these increases in expression of iNOS and STAT‐1 phosphorylation were significantly suppressed by the administration of PI, which is an agonist of PPARγ (Figure 1A–E). Furthermore, IL‐4/IL‐13 was added to induce M2 macrophage polarization. Stimulation with IL‐4/IL‐13 significantly increased the expression of Arg‐1, Fizz 1, Ym 1, and STAT‐6 phosphorylation. We used SR202 to inhibit the expression of PPARγ and found that the increases in expression of Arg‐1, Fizz 1, Ym 1, and STAT‐6 phosphorylation were significantly suppressed (Figure 1F–L). To further investigate the effect of PPARγ on macrophage polarization, LPS/IFN‐γ was added to M2 macrophages with or without PI. After M2 macrophages were subjected to LPS/IFN‐γ treatment, the expression of iNOS and phosphorylated STAT‐1 significantly increased, and the expression of Arg‐1, Fizz 1, Ym 1, and phosphorylated STAT‐6 significantly decreased. However, the increases in the expression of iNOS and STAT‐1 phosphorylation were suppressed, whereas the expression of Arg‐1, Fizz 1, Ym 1, and STAT‐6 phosphorylation increased with the administration of PI (Figure 2). These results indicated that the activation of PPARγ could inhibit the repolarization of macrophages from the M2 phenotype to the M1 phenotype and maintain the M2‐like phenotype.

### 3.2 PPARγ regulated the STAT‐1/STAT‐6 pathway to induce the polarization of macrophages from the M1 to the M2 phenotype in vivo

**FIGURE 3:**
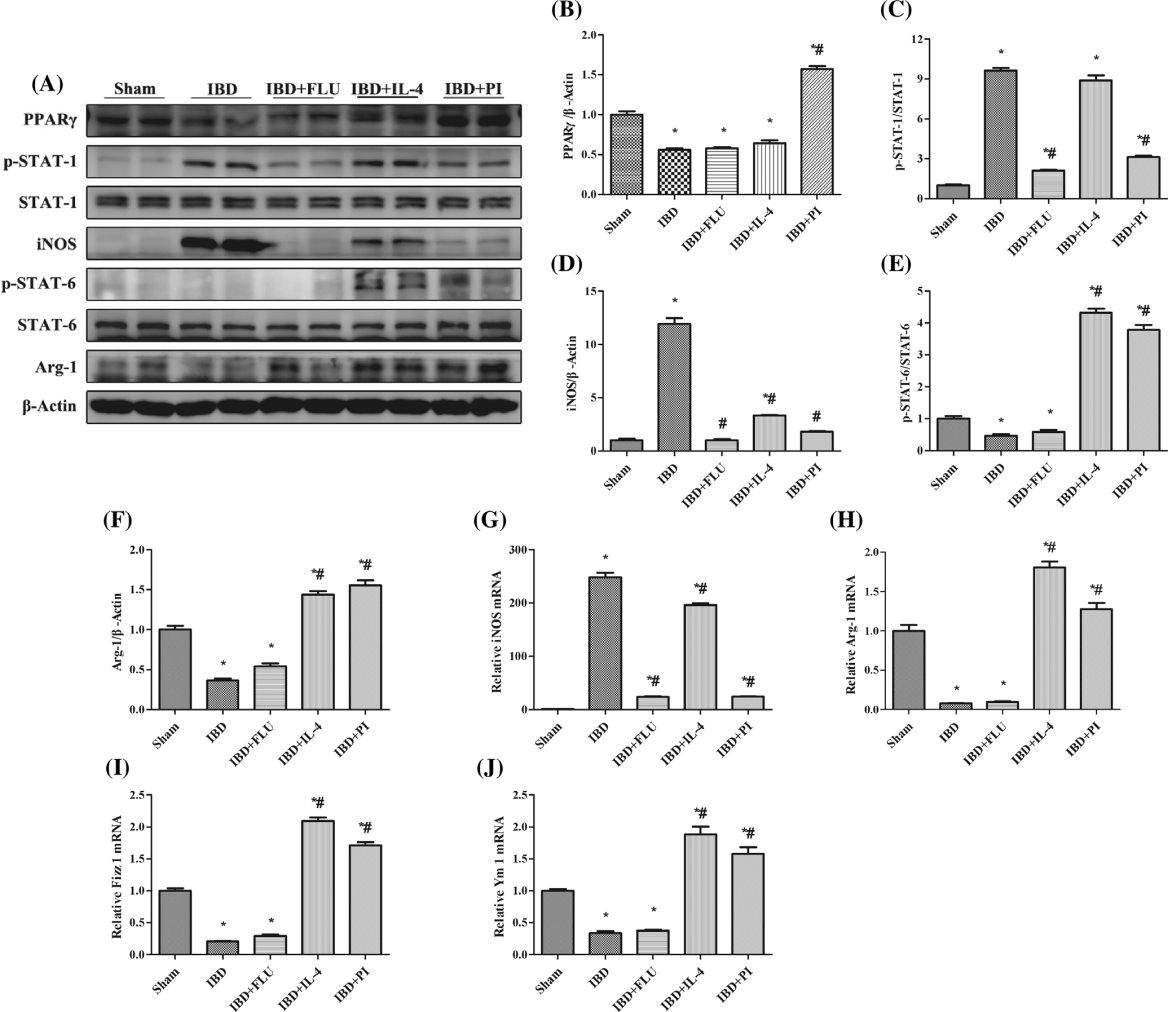
PPARγ regulated the STAT‐1/STAT‐6 pathway to induce the polarization of macrophages from M1 to M2 in vivo. The protein expression of PPARγ, p‐STAT‐1, STAT‐1, iNOS, PPARγ, p‐STAT‐6, STAT‐6, and Arg‐1 (A) was measured by western blotting. The relative protein expression of PPARγ (B), iNOS (D), and Arg‐1 (F) was normalized to that of β‐Actin. The protein expression of p‐STAT‐1 (C) was normailzed to that of STAT‐1. The protein expression of p‐STAT‐6 (E) was normailzed to that of STAT‐6. The mRNA expression of iNOS (G), Arg‐1 (H), Fizz 1 (I), and Ym 1 (J) was measured by real‐time polymerase chain reaction. The expression of each mRNA was normalized to that of β‐Actin. The data are presented as the mean ± standard error of the mean. *p < 0.05 versus the Sham group. ^#^ *p* < 0.05 versus the IBD group.

**FIGURE 4:**
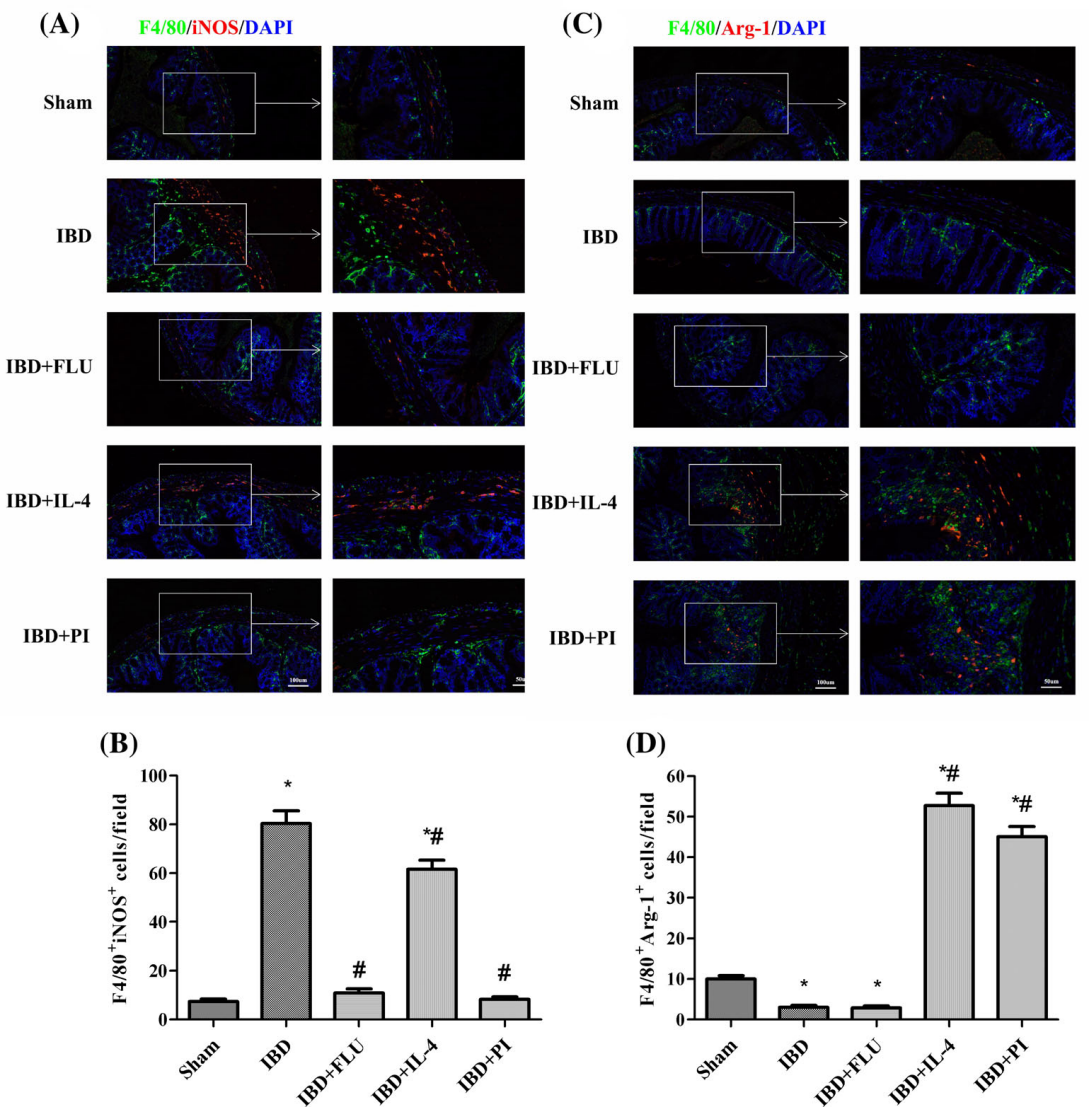
Activation of PPARγ modulated the plasticity of intestinal macrophages. Immunofluorescence images of F4/80 (green), iNOS (red), and DAPI (blue) in the colon (A). The number of F4/80^+^ iNOS^+^ cells were quantified (B). Immunofluorescence images of F4/80 (green), Arg‐1 (red), and DAPI (blue) in the colon (C). The number of F4/80^+^ Arg‐1^+^ cells were quantified (D).

As shown in Figure 3, compared with the Sham group, DSS treatment significantly increased the expression of iNOS and decreased the expression of Arg‐1, Fizz 1, and Ym 1, which indicated that colonic macrophages had polarized toward the M1 phenotype in mice with DSS‐induced IBD. We also found that DSS treatment increased STAT‐1 phosphorylation and decreased STAT‐6 phosphorylation. When FLU was used to inhibit STAT‐1 phosphorylation, the expression of iNOS significantly decreased with no notable changes in Arg‐1, Fizz 1, or Ym 1 expression. Similarly, we used IL‐4 to activate STAT‐6 phosphorylation and found that the expression of Arg‐1, Fizz 1, and Ym 1 significantly increased. These results indicated that the inhibition of STAT‐1 phosphorylation could suppress the M1 polarization of macrophages and that the activation of STAT‐6 phosphorylation could promote the M2 polarization of macrophages without changing PPARγ expression. Moreover, the administration of PI decreased the expression of iNOS and increased the expression of Arg‐1, Fizz 1, and Ym 1 by inhibiting STAT‐1 phosphorylation and promoting STAT‐6 phosphorylation. Immunofluorescence double‐staining was performed to further detect whether activation of PPARγ affects the quantity of M1 and M2 macrophages in the colon tissues of mice with IBD. F4/80 macrophages were concentrated mainly in the intestinal lamina propria. The number of F4/80^+^ iNOS^+^ M1 macrophages decreased with the administration of PLU, and the number of F4/80^+^ Arg‐1^+^ M2 macrophages increased with the administration of IL‐4. The administration of PI significantly reduced the number of F4/80^+^ iNOS^+^ M1 macrophages and increased the number of F4/80^+^ Arg‐1^+^ M2 macrophages in the colon tissues of mice with IBD (Figure 4). These results indicated that the activation of PPARγ could induce the polarization of macrophages from the M1 phenotype to the M2 phenotype via the STAT‐1/STAT‐6 pathway in mice with DSS‐induced IBD.

### 3.3 Activation of PPARγ attenuated DSS‐induced IBD in mice

**FIGURE 5:**
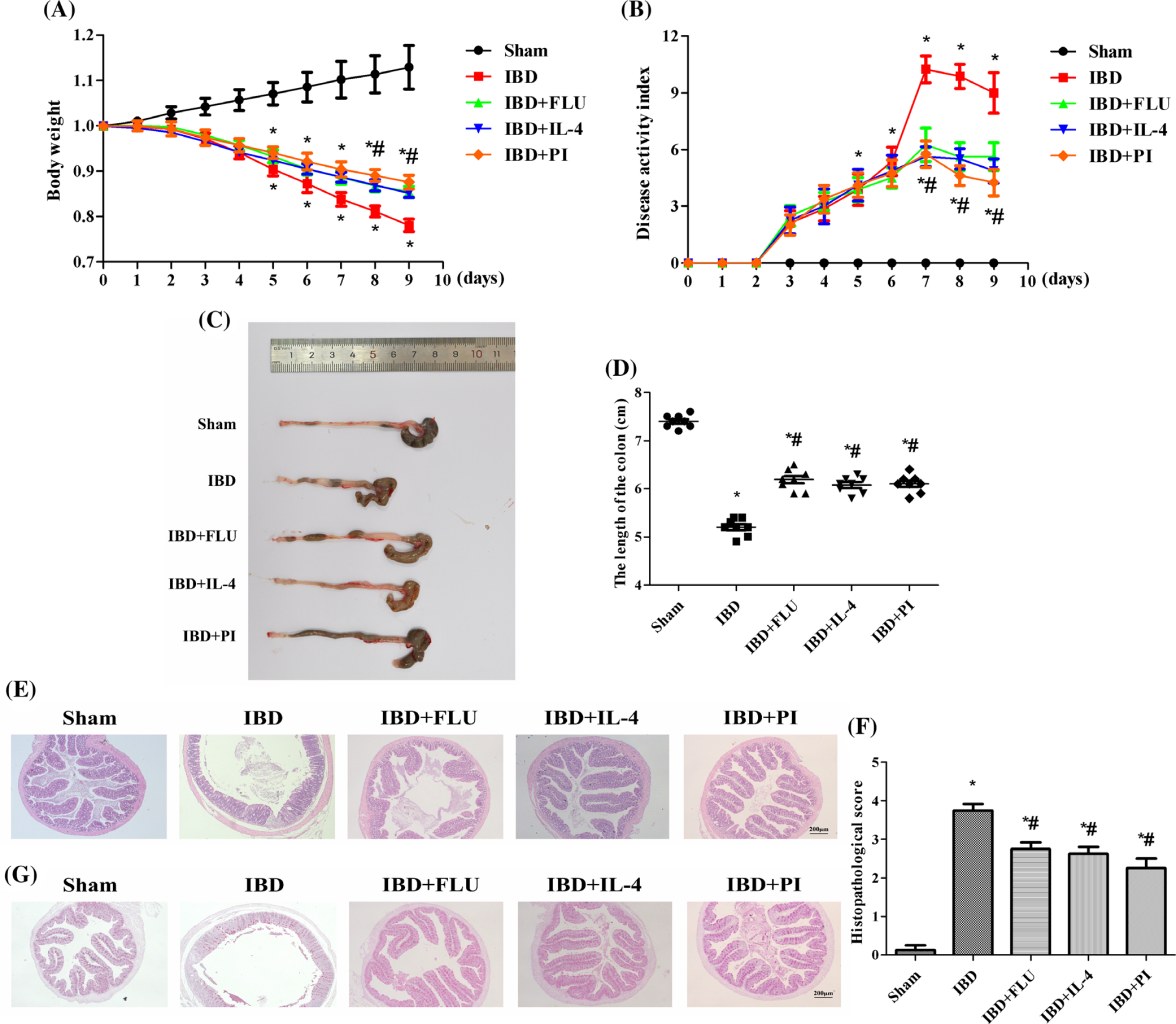
Activation of PPARγ attenuated DSS‐induced IBD in mice. The weight of mice was assessed daily (A). The evaluation of disease activity index score of mice (B). Representative pictures of colon (C) and the length of the colon were measured (D). Histologic changes in the colon were evaluated with hematoxylin–eosin (H&E) staining (E). Histopathologic scoring of the colon (F). The goblet cell density and mucosal surface in the colon were evaluated by PAS staining (G). The data are presented as the mean ± standard error of the mean. *p < 0.05 versus the Sham group. ^#^ *p* < 0.05 versus the IBD group.

**FIGURE 6:**
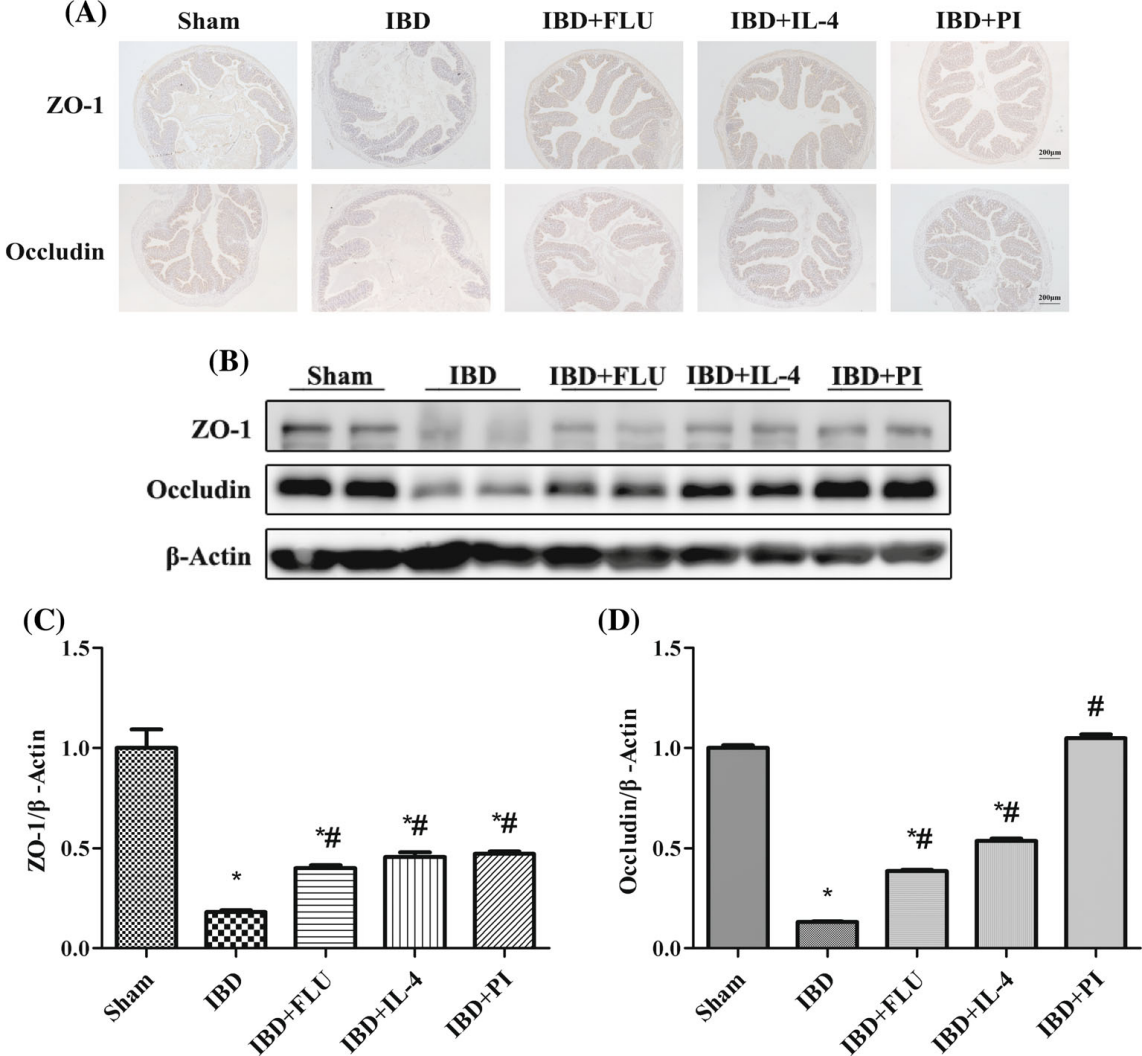
Activation of PPARγ attenuated intestinal barrier dysfunction. The expression of ZO‐1 and occludin in the colon was evaluated by immunofluorescence (A) and western blotting (B). The relative protein expression of ZO‐1 (C) and occludin (D) was normalized to that of β‐Actin. The data are presented as the mean ± standard error of the mean. *p < 0.05 versus the Sham group. ^#^ *p* < 0.05 versus the IBD group.

The weights of the mice significantly decreased after DSS treatment (Figure 5A). DSS also caused severe colonic injury, including diarrhea, bloody stool, and atrophy of the colon (Figure 5B–D). H&E‐stained colon sections revealed inflammatory cell infiltration in the lamina propria and disordered tissue structure (Figure 5E) in the IBD group. PAS revealed that DSS reduced the density of goblet cells and the mucosal surface area (Figure 5G). In contrast, in the IBD group, the administration of PI reversed the weight loss and colonic atrophy and reduced the DAI score. PI also reduced inflammatory cell infiltration in the lamina propria and the histopathological score and restored the density of goblet cells and the mucosal surface area (Figure 5). Moreover, intestinal barrier function was also measured by immunohistochemistry and western blotting. DSS treatment decreased the expression of ZO‐1 and occludin. The administration of PI reversed the DSS‐induced changes in the expression of ZO‐1 and occludin, which indicated that the activation of PPARγ had a protective effect on intestinal barrier function (Figure 6).

## 4 DISCUSSION

The present study aimed to investigate the role of PPARγ in the regulation of macrophage polarization and its potential therapeutic effects on DSS‐induced IBD in mice. Our findings demonstrated that the activation of PPARγ could regulate M1/M2 macrophage polarization via the STAT‐1/STAT‐6 pathway both in vitro and in vivo, thereby attenuating the symptoms of IBD.

At present, the treatment of IBD, which includes 5‐ASA, mesalazine, balsalazide, corticosteroids, anti‐inflammatory agents, and antibiotic therapy, mainly promotes epithelial proliferation and barrier integrity by restoring microbial homeostasis and inhibiting immune responses. However, many patients still need surgery to remove damaged parts of the intestine, and many drugs still need to be optimized. Therefore, new treatment methods are needed to counteract the destruction of mucosa and submucosal tissue and avoid the side effects of long‐term 5‐ASA and corticosteroid treatment in IBD patients.^13^ As key cells that regulate the innate immune system, macrophages are crucial for regulating intestinal homeostasis and inflammation.^4^ Macrophages can shift their phenotype to either a proinflammatory or a proresolving phenotype, depending on diverse stimuli and unique cellular microenvironments. In the inflamed intestine, macrophages polarize toward the M1 phenotype and secrete proinflammatory cytokines, leading to the development of IBD. In addition to a significant increase in M1 macrophage numbers, patients with IBD also exhibit a decrease in M2 macrophage numbers.^23^, ^24^ STAT‐1 is an important transcription factor associated with M1 macrophages, and STAT‐6 is an important transcription factor associated with M2 macrophages.^7^, ^10^ The traditional herb astragaloside IV can inhibit macrophage M1 polarization to alleviate symptoms in mice with DSS‐induced colitis by downregulating STAT‐1.^25^ The Chinese medicine Wumeiwan can promote macrophage M2 polarization to decrease the expression of proinflammatory cytokines by activating the STAT‐6 pathway.^26^ In this study, we used FLU to inhibit M1 polarization by inhibiting STAT‐1 phosphorylation, and we used IL‐4 to promote M2 polarization by promoting STAT‐6 phosphorylation; we found that inhibiting M1 polarization or promoting M2 polarization could significantly attenuate IBD symptoms.

**FIGURE 7:**
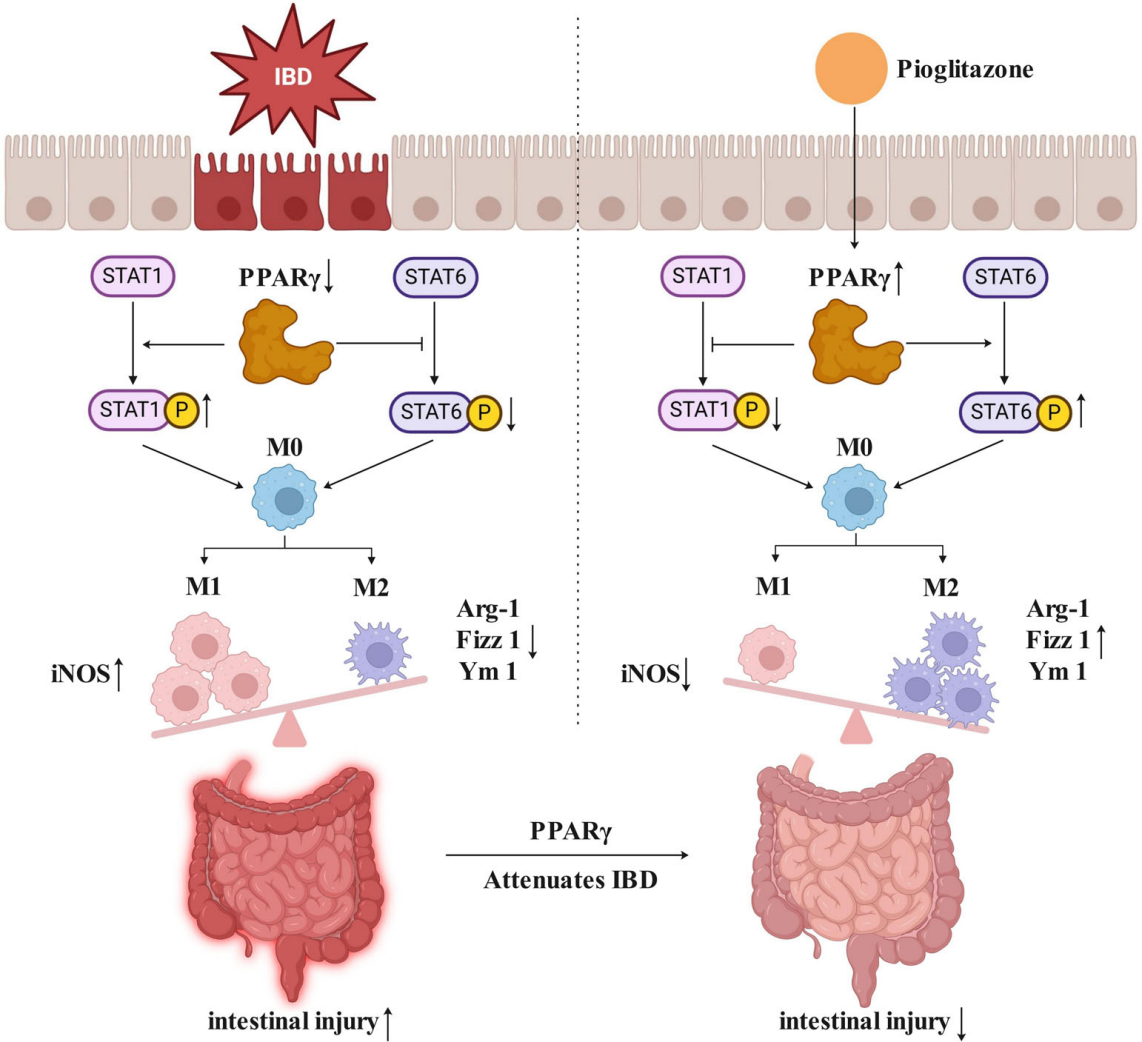
Mechanism diagram of activation of PPARγ affecting macrophage polarization and IBD.

PPARγ is a nuclear receptor transcription factor. In addition to regulating carbohydrate and lipid metabolism and energy balance, PPARγ has recently been shown to maintain normal intestinal homeostasis. The expression of PPARγ in the colonic epithelium of UC patients is lower than that in control subjects.^27^ Butyrate‐mediated PPARγ activation restores the balance of Treg/Th17 cells to attenuate IBD.^28^, ^29^ Long‐chain n‐3 polyunsaturated fatty acids can suppress inflammatory signaling by activating PPARγ to reduce the incidence of IBD.^30^ Treatment with moringin increases the expression of PPARγ to protect against IBD by improving intestinal barrier function.^31^ The administration of a PPARγ selective agonist (GED 0507‐34 Levo) prevents the aberrant induction of the epithelial–mesenchymal transition, fibrogenesis, and senescence in DSS‐treated mice.^32^ A multicenter, randomized, double‐blind, placebo‐controlled clinical trial revealed that the use of rosiglitazone (4 mg, orally administered twice daily), a thiazolidinedione ligand for PPARγ, effectively alleviated mild to moderate active IBD.^33^ Rosiglitazone (4 mg, enema treatment once daily for 14 days) increased the expression of PPARγ in the inflamed colonic epithelium and exerted local anti‐inflammatory effects in the guts of patients with IBD.^34^ In this study, we also revealed that DSS caused severe colonic injury and decreased PPARγ expression. A PPARγ agonist could rescue embryo absorption by inhibiting M1 macrophage polarization.^35^ The inhibition of PPARγ expression by translocator protein ligands inhibits IL‐4‐induced M2 polarization at the site of hypoxic ischemia in the brain.^36^ Activation of PPARγ induced by 15d‐PGJ2 polarizes macrophages from the M1 phenotype to the M2 phenotype, which can suppress cytokine release and neutrophil migration.^37^ Inflammatory macrophages can be polarized toward the M2 phenotype to eliminate atherosclerosis by increasing the expression of PPARγ, and preventing PPARγ activation significantly impairs macrophage phagocytosis.^38^ Macrophage‐specific PPARγ deficiency significantly increases the expression of inflammatory and metabolic genes and both the infiltration and activation of macrophages and T cells in the colonic mucosal lamina propria, which exacerbates the clinical activity and colonic pathology of IBD.^39^ In vitro experiments using the RAW264.7 macrophage line demonstrated that activation of PPARγ by PI decreased M1 polarization marker expression and STAT‐1 phosphorylation. Moreover, activation of PPARγ increased the expression of M2 polarization markers, including Arg‐1, Fizz 1, and Ym 1, and increased STAT‐6 phosphorylation. These results indicated that the activation of PPARγ could inhibit the repolarization of macrophages from the M2 phenotype to the M1 phenotype and maintain the M2 phenotype. Consistent with the in vitro findings, our in vivo experiments in mice with DSS‐induced IBD revealed that the activation of PPARγ could attenuate disease symptoms, such as weight loss, diarrhea, and bloody stool. Histological analysis revealed that PI treatment reduced inflammatory cell infiltration, restored the mucosal architecture, and improved the expression of tight junction proteins, all of which indicate an improvement in intestinal barrier function. Moreover, our study demonstrated that the activation of PPARγ could modulate the polarization of colonic macrophages from the M1 phenotype to the M2 phenotype via the STAT‐1/STAT‐6 pathway. In mice with DSS‐induced IBD, the expression of M1 markers was increased, whereas the expression of M2 markers was decreased. Treatment with PI reversed these changes by inhibiting STAT‐1 phosphorylation and promoting STAT‐6 phosphorylation, leading to a reduction in M1 macrophage numbers and an increase in M2 macrophage numbers (Figure 7). Together, these findings suggest that the activation of PPARγ may represent a novel therapeutic strategy for IBD and that this approach may have therapeutic effects by restoring the balance of macrophage polarization and reducing excessive inflammation in the intestinal mucosa.

This study has certain limitations. First, the use of a single animal model (DSS‐induced colitis model) may not fully capture the complexity of IBD. Further studies in additional models and patients with IBD could strengthen the findings. Second, we confirmed that the activation of PPARγ could inhibit the repolarization of macrophages from the M2 phenotype to the M1 phenotype and maintain the M2 phenotype in RAW264.7 cells and colon tissues to attenuate IBD. However, whether this effect is entirely dominated by macrophages or whether other cells, such as neutrophils or T cells, also participate requires further investigation.

In conclusion, our study demonstrated that the activation of PPARγ plays a crucial role in regulating macrophage polarization and has therapeutic potential in the treatment of IBD. By promoting M2 polarization and inhibiting M1 polarization via the STAT‐1/STAT‐6 pathway, the activation of PPARγ can attenuate the symptoms of IBD and restore intestinal barrier function. These findings provide a novel target for the development of therapeutic strategies for IBD and highlight the importance of macrophage polarization in the pathogenesis of IBD. Further study is needed to explore the potential of numerous recently developed PPARγ‐selective agonists for use in the clinical treatment of IBD.

## CONFLICT OF INTEREST STATEMENT

The authors declare no conflict of interest.

## Data Availability

Data are available from the corresponding author upon reasonable request.
